# Detection of in vivo mutagenicity in rat liver samples using error-corrected sequencing techniques

**DOI:** 10.1186/s41021-023-00288-z

**Published:** 2023-11-22

**Authors:** Kazuki Izawa, Masataka Tsuda, Takayoshi Suzuki, Masamitsu Honma, Kei-ichi Sugiyama

**Affiliations:** 1 Division of Genetics and Mutagenesis, National Institute of Health Sciences, 3-25-26, Tonomachi, Kawasaki-ku, Kawasaki-shi, Kanagawa 210-9501 Japan; 2 Division of General Affairs, National Institute of Health Sciences, 3-25-26, Tonomachi, Kawasaki-ku, Kawasaki-shi, Kanagawa 210-9501 Japan

**Keywords:** In vivo mutagenicity, Next-generation sequencing, Error-corrected sequencing, Mutational signatures, Rat liver sample

## Abstract

**Background:**

Mutagenicity, the ability of chemical agents to cause mutations and potentially lead to cancer, is a critical aspect of substance safety assessment for protecting human health and the environment. Metabolic enzymes activate multiple mutagens in living organisms, thus in vivo animal models provide highly important information for evaluating mutagenicity in human. Rats are considered suitable models as they share a similar metabolic pathway with humans for processing toxic chemical and exhibit higher responsiveness to chemical carcinogens than mice. To assess mutagenicity in rats, transgenic rodents (TGRs) are widely used for in vivo gene mutation assays. However, such assays are labor-intensive and could only detect transgene mutations inserted into the genome. Therefore, introducing a technology to directly detect in vivo mutagenicity in rats would be necessary. The next-generation sequencing (NGS) based error-corrected sequencing technique is a promising approach for such purposes.

**Results:**

We investigated the applicability of paired-end and complementary consensus sequencing (PECC-Seq), an error-corrected sequencing technique, for detecting in vivo mutagenicity in the rat liver samples. PECC-Seq allows for the direct detection of ultra-rare somatic mutations in the genomic DNA without being constrained by the genomic locus, tissue, or organism. We tested PECC-Seq feasibility in rats treated with diethylnitrosamine (DEN), a mutagenic compound. Interestingly, the mutation and mutant frequencies between PECC-Seq and the TGR assay displayed a promising correlation. Our results also demonstrated that PECC-Seq could successfully detect the A:T > T:A mutation in rat liver samples, consistent with the TGR assay. Furthermore, we calculated the trinucleotide mutation frequency and proved that PECC-Seq accurately identified the DEN treatment-induced mutational signatures.

**Conclusions:**

Our study provides the first evidence of using PECC-Seq for in vivo mutagenicity detection in rat liver samples. This approach could provide a valuable alternative to conventional TGR assays as it is labor- and time-efficient and eliminates the need for transgenic rodents. Error-corrected sequencing techniques, such as PECC-Seq, represent promising approaches for enhancing mutagenicity assessment and advancing regulatory science.

## Background

Mutagenicity refers to the potential of chemical agents to cause mutations and potentially leading to cancer development. Hence, evaluating mutagenicity is a crucial aspect of chemical safety assessment, aimed at safeguarding human health and the environment [1]. Multiple chemical mutagens and carcinogens are mutagenic as they are converted into mutagens by metabolic enzymes in vivo. In addition, the tissue affected by toxicity might differ from where activation takes place as metabolites could be transported to different target tissues [2]. Therefore, in vitro models cannot fully replicate all the metabolic and distribution complexities of in vivo exposures, evaluating mutagenicity in an in vivo animal model would be required.

Rats are considered more similar to humans than mice as they share a similar metabolic pathway to humans for processing chemicals [3, 4]. In addition, the guidelines of the International Council for Harmonization of Technical Requirements for Pharmaceuticals for Human Use (ICH) suggest using rats as the standard animal species for testing the carcinogenicity of pharmaceuticals [5]. This recommendation relies on the fact that rats are more responsive to chemical carcinogens than mice, and positive results observed in the liver of mice might not necessarily prove relevant to human carcinogenic risks. Indeed, the rats are the preferred animal in general toxicity tests such as the 28-day short-term toxicity test. Therefore, rat-based in vivo mutagenicity tests hold an important position in regulatory science.

In vivo gene mutation assays utilizing transgenic rodents (TGR) represents a promising approach to assess in vivo mutagenicity, as they reflect whole-organism biology. The TGR assay uses transgenic rats and mice carrying multiple copies of chromosomally integrated plasmids or phage shuttle vectors containing reporter genes. The assays enable the scoring of mutations induced in a transgene in any tissue of the rodent [6–8]. Assessing mutational spectra from TGR assays involves manually selecting hundreds of *Escherichia coli* colonies or phage plaques for sequencing, this approach is thus highly labor-intensive and time-consuming. In addition, the TGR assay only detects mutations on transgenes inserted into the genome; however, evidence showing to indicate that it perfectly reflects mutations occurring in the whole genome is insufficient. Therefore, introducing a technology for direct mutation detection in the genome, not in the transgene, would be highly desired.

The potential to directly detect ultra-rare somatic mutations from the extracted DNA without being constrained by the genomic locus, tissue, or organism is highly attractive. Next-generation sequencing (NGS) is an advanced technology for determining DNA sequences rapidly and with high throughput. However, the NGS technical error rate (∼1.0 × 10^−3^) is significantly higher than the true nucleotide mutation frequency of normal tissues (< 1.0 × 10^−7^) [9, 10]. Recently, various error-corrected next-generation sequencing techniques (ecNGS) have been developed using the NGS technology, aiming at detecting rare somatic mutations from genomic samples derived from a bulk of somatic cells [9–14]. ecNGS uses the concept of consensus sequencing of DNA fragments coupled with bioinformatics to eliminate sequencing errors, leading to error rates below one per one million sequenced bases [15]. There is difference between using unique molecular identifiers or not, the common strategy of ecNGS techniques increases read redundancy from a unique DNA molecule and the error tolerance of each base in the reads [9–15]. These techniques have also been considered important and applied for detecting in vivo mutagenicity in mice [13, 16]. Beyond the labor- and time-efficiency of the ecNGS techniques compared to the TGR assay, they also display the potential to detect in vivo mutagenicity without transgenic rodents. This means that animal samples from other toxicological assays can be shared to detect in vivo mutagenicity. This also enables a great reduction in animal testing and contributes to the 3Rs of animal testing. Thus, the ecNGS assay results from rat samples are an urgent need for developing a multi-endpoint assay in rats.

We previously developed paired-end and complementary consensus sequencing (PECC-Seq), which is an ecNGS technique that uses a simple modified PCR-free sequencing process to enhance consensus creation efficacy from whole genome sequencing data [10]. Both library preparations and subsequent bioinformatic analysis were further streamlined. We successfully detected in vitro mutagenicity using the TK6 human cell line with PECC-Seq [10].

In this study, we tested the feasibility of in vivo mutagenicity measurements in rat liver samples using PECC-Seq. We assessed the genomic DNA of *gpt* delta rats treated with diethylnitrosamine (DEN), a mutagenic compound in the liver, and analyzed mutational signatures. Consistent with the TGR assay, PECC-Seq detected the A:T > T:A mutation in the investigated rat liver samples. To the best of our knowledge, this study is the first to demonstrate the detection of in vivo mutagenicity in rat liver samples using PECC-Seq.

## Results and discussions

The F344/Nslc *gpt* delta rat strain is used for TGR assays. In previous study, the rats were DEN-treated once a week for 5 weeks. In the *gpt* assays, approximately 120-fold increase in mutant frequency was observed in the livers of DEN-treated rats [17]. We conducted PECC-Seq on rat liver samples using non-treated control (Control 3, 8, and 9) and DEN-treated (DEN P11, P13, and P15) group of the above-described samples.

**Fig. 1:**
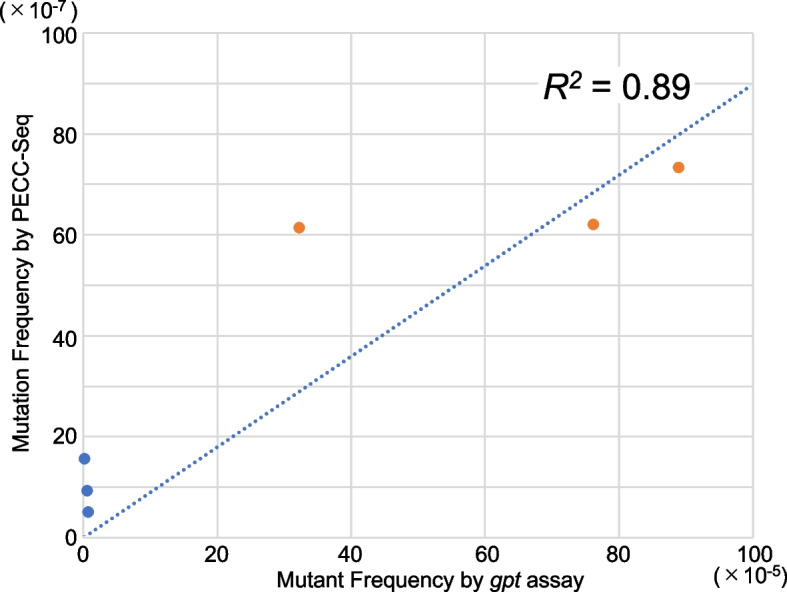
The correlation of mutant frequencies from the *gpt* assay and mutation frequencies from PECC-Seq analysis. The X-axis indicates the mutant frequency using the *gpt* assay in a 10^−5^ order, whereas the Y-axis indicates the mutation frequency using PECC-Seq in a 10^−7^ order for each sample. The blue and orange dots represent the non-treated control and DEN-treated samples, respectively. The dashed line indicates the linear regression line of the mutant frequency using the *gpt* assay and the mutation frequency using PECC-Seq

We measured 1.00 × 10^−6^ and 6.56 × 10^−6^ in the non-treated control and DEN-treated groups, respectively, as average mutation frequencies using PECC-Seq (Table 1). Our statistical analysis demonstrated that the mutation frequency of the two groups differed significantly (*p* < 0.05). We could thus conclude that PECC-Seq successfully detected the mutations introduced by the DEN treatment. However, the sensitivity of the PECC-Seq in rat is not satisfactory compared with the fold increase of mutant frequencies in the *gpt* assay. Because PECC-Seq does not directly use SNP information of animals, using a small number of animals may affect false positive mutation detection derived from animal-specific SNPs. Further analysis of animals or SNP information accumulation of this strain could help eliminate mutations detected as false positives in non-treated animals. The coefficient of variation (CV) of the mutation frequencies between the samples in each group is as follows: For the non-treated samples, the CV was 0.60 and 0.53 for the *gpt* assay mutant frequency and PECC-Seq mutation frequency, respectively (Table 1). For DEN-treated samples, the CV was 0.45 and 0.10 for the *gpt* assay mutant frequency and PECC-Seq mutation frequency, respectively (Table 1). Although the PECC-Seq analysis showed a slightly high CV value in non-treated group due to false positive mutation detection derived from animal-specific SNPs, which we cannot fully exclude, the robustness of PECC-Seq for chemical-induced mutation detection is indicated. The correlation (*R*^*2*^ = 0.89) between the mutant frequencies from the *gpt* assay and mutation frequencies from PECC-Seq was promising, especially considering that these assays detect mutation frequencies using two fundamentally distinct approaches (Fig. 1).

**Table 1:** Summary of PECC-Seq analysis and mutation frequencies of the analyzed samples

Sample name	Number of Reads	Analyzed bases	Number of Mutations	Mutation frequencies (× 10^−6^)	*gpt* assay Mutant frequencies^a^ (× 10^−5^) [17]
Control 3	987,423,042	38,838,583	20	0.51	0.70
Control 8	1,077,081,792	37,591,524	35	0.93	0.52
Control 9	1,065,771,452	38,233,853	60	1.57	0.16
DEN P11	923,538,038	64,822,157	476	7.34	88.89
DEN P13	886,468,356	60,749,075	373	6.14	32.24
DEN P15	812,319,594	56,400,757	350	6.21	76.19

^a^*gpt* assay mutant frequencies reported in previous study [17]

**Fig. 2:**
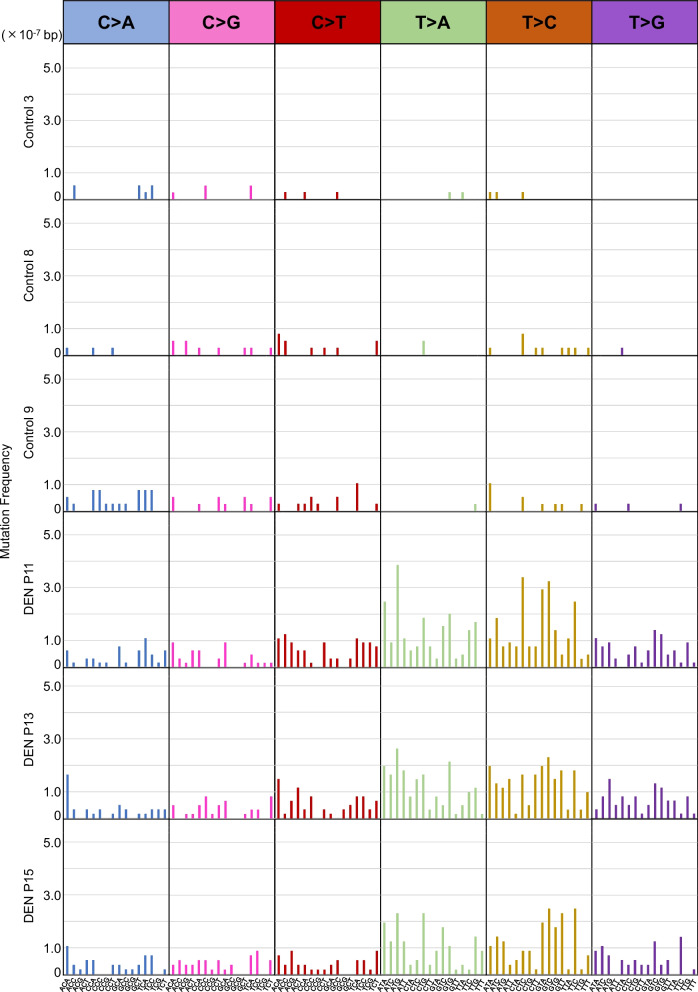
Trinucleotide mutational signatures of rat liver samples from the non-treated control (Control 3, 8, and 9) and DEN-treated (DEN P11, P13, and P15) groups. The X-axis indicates each trinucleotide pattern in each mutation pattern column. The Y-axis indicates the mutation frequency in a 10^−7^ order in each sample row

**Fig. 3:**
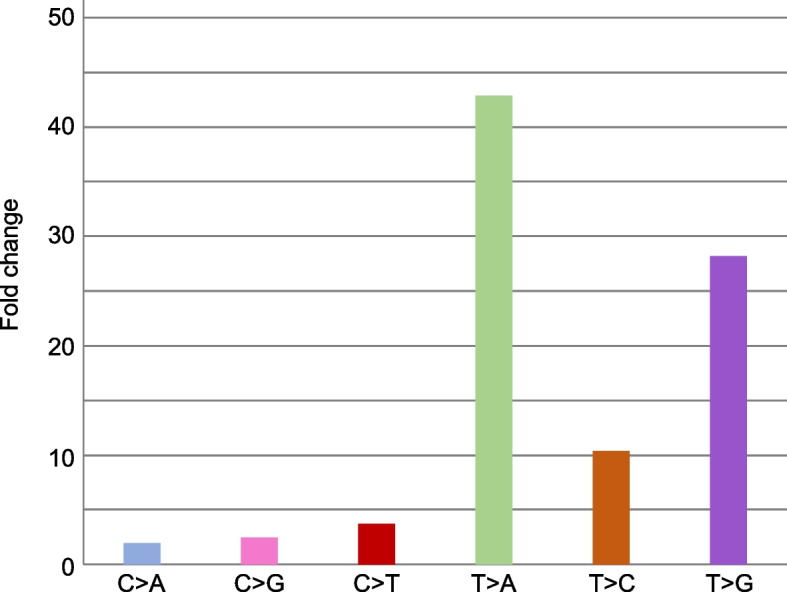
The fold changes of the average mutation pattern frequencies between the non-treated control and DEN-treated groups. The Y-axis indicates the fold change. Bar plots indicate the fold change of each mutation pattern

**Fig. 4:**
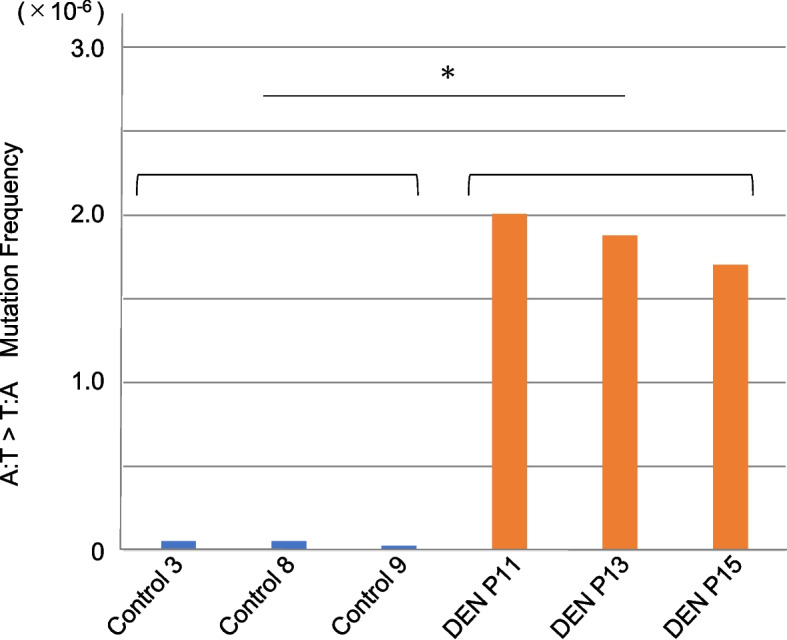
The mutation frequency of A:T > T:A in the non-treated control and DEN-treated group samples (blue and orange bars, respectively). The Y-axis indicates the A:T > T:A mutation frequency in a 10^−6^ order in each sample. * indicates *p* < 0.05

Next, to confirm the mutational signature of DEN-treatment, we analyzed the trinucleotide base substitution spectra in the non-treated control and DEN-treated group samples. The samples from each group showed a highly similar mutational signature (Fig. 2). These results also indicate the robustness of PECC-Seq for in vivo mutational signature detection. The robustness of ecNGS for in vivo mutagenicity detection was discussed in a previous study [13]. PECC-Seq also showed a robust in vivo mutation detection performance. Our results also revealed high A:T > T:A mutation in the DEN-treated group (Fig. 3). Consistently, the mutation spectrum introduced by DEN in the *gpt* gene corresponds to the A:T > T:A transversion mutation [18]. The average A:T > T:A mutation frequency in the non-treated control and DEN-treated groups were 4.36 × 10^−8^, and 1.87 × 10^−6^, respectively (representing approximately 43-fold increase compared to the non-treated control) (Fig. 4). The statistical test also showed that A:T > T:A mutation frequency is significantly high in DEN-treated group samples.

Following the A:T > T:A transversion mutation, A:T > C:G transversion and A:T > G:C transition mutation were highly introduced in DEN-treated group (Fig. 3). In contrast, the DEN-introduced mutation spectra in the *gpt* gene reportedly comprises the A:T > T:A transversion mutation, followed by the G:C > A:T and A:T > G:C transition mutation [18]. Namely, inconsistency could be identified in the second and third frequent mutation spectra between this and previous study. Several mechanisms could explain this apparent difference. One of the explanations is frequent amplifiable template lesions occur during DNA extraction and preparation, linked to higher oxidation and hydrolytic deamination rates [19, 20]. For instance, Costello et al. described increased G > T transversions being directly connected to DNA preparation protocols that cause 8-oxoguanine (8-oxoG) lesions, a common oxidative DNA damage result [21]. The *gpt* assay procedure comprises multiple DNA handling steps to detect mutations as *E. coli* colonies [8] and also the *gpt* gene packaging efficiency of the F344/Nslc *gpt* delta rat strain is low due to the low copy number of the *gpt* gene integrated in genome [22]. DNA oxidation and hydrolytic deamination might also be introduced during the various experimental steps and labor-intensive way of detecting mutation. Another possible reason to explain the mutational difference is the GC content difference as the contents of the *gpt* gene and the rat genome are 51.9 and 41.5%, respectively. Moreover, the nucleotide ratio could also affect the mutation spectra. Taken together, transgene-specific assays might confer potential biases to the mutational spectrum and does not provide sufficient mutational information, which could perfectly reflect mutations in the entire genome. We thus favor the idea of direct and genome-wide mutation detection, although not in a locus- and transgene-specific manner.

In conclusion, we demonstrated that PECC-Seq could detect in vivo mutagenicity in rat liver samples along with a characteristic mutational signature induced by DEN. Taking into account all abovementioned considerations, ecNGS techniques represented by PECC-Seq could provide useful tools for detecting in vivo mutagenicity as these techniques require no transgenic rodents and save time and labor compared to TGR assay. Moreover, our results indicates that ecNGS can detect in vivo mutagenicity using samples from other toxicological assays and contribute to the 3R of animal research.

## Materials and methods

### Genomic DNA extraction from rat liver samples and NGS library preparation

F344/Nslc *gpt* delta male rat liver samples were obtained from a previous study [17], provided by the Division of Pathology, National Institute of Health Sciences. For the non-treated control, corn oil was intragastrically administrated to the animals once daily for 13 weeks. For the DEN treatment, 40 mg/kg body weight of DEN was intraperitoneally administrated to the animals once a week for 5 weeks. Genomic DNA was extracted using the DNeasy Blood & Tissue Kits (QIAGEN, Venlo, Nederland) following the manufacturer’s protocol. The sequencing libraries were prepared mostly following a previous report [10] with additional steps. Briefly, after the genomic DNA fragmentation by sonication, the fragmented DNA samples were subjected to S1 nuclease (Thermo Scientific™, MA, USA) treatment following the manufacturer’s protocol in order to reduce artificial mutations [20]. Approximately 150-bp DNA fragments were subjected to constructing Illumina sequencing libraries using the TruSeq DNA PCR-Free Kit (Illumina, Inc., CA, USA).

### Sequencing and analysis

The obtained sequence libraries were sequenced using the Illumina NextSeq 2000 platform (Illumina, Inc., CA, USA) with the NextSeq 1000/2000 P2 (300 cycle) or P3 (300 cycle) reagents. The libraries were diluted to 50 pM as final loading concentration. The information processing mostly followed a previous study [10]. Briefly, the adapter sequences in raw sequencing data were trimmed by Trimmomatic (v0.39) [23] with following command.

java -jar trimmomatic-0.39.jar PE -threads 16 -phred33 read_1.fastq read_2.fastq read_out_1.fastq read_out_2.fastq ILLUMINACLIP: TruSeq3-PE-2.fa:2:30:10:1:true

The library terminal end trim was not applied in this study because the S1 nuclease treatment eliminated the artificial mutations at the terminal regions. After adapter sequence trimming, the paired-end reads were mapped on to F344/NHsd rat genome sequence [24] downloaded from GigaDB (http://gigadb.org/dataset/100042) using the Burrows-Wheeler aligner (BWA) [25] in default setting of “bwa mem” mode. Mapping results were sorted by SAMtools (v1.9) [26] and properly mapped paired-end reads with high quality score (more than 60) were extracted.

To obtain the consensus read groups, firstly we randomly extracted 10% data by using list of 5′ end location of all mapped reads for the reason of computational resource. After the 10% data extraction, only four reads share the same mapped location with proper orientation (paired-end reads with complementary strand) were grouped as consensus read group. The mapping data with consensus read groups were extracted and mutations suggested by consensus read groups were detected by SAMtools.

To exclude animal SNPs, mutations detected from all mapping data by SAMtools were extracted. The mutations suggested by all mapping data were excluded from the mutations suggested by consensus read groups. These candidate mutations suggested by consensus read groups were confirmed with the IGV browser [27]. Firstly, these candidates were filtered with supporting read depth. The positions of mutation with read depth calculated from all mapped reads more than 30 reads and less than 100 reads were extracted. Then, consensus read groups with more than 2 mutations were discarded. Additionally, mutations that also supported by other reads not in the consensus read group were discarded for exclude the animal-specific SNPs. These filtered mutations were considered as chemical-induced mutation and subjected to following analysis.

### Statistical analysis

The average mutation frequency was calculated as the sum of mutations in each group divided by the sum of PECC-Seq-analyzed bases in each group. All mutations detected by PECC-Seq were classified to each trinucleotide type. The trinucleotide frequency was calculated as the sum of mutations in each type divided by the sum of the PECC-Seq-analyzed bases. The fold change of mutations in each mutation group was calculated as the sum of the mutations in each mutation group of the DEN-treated group divided by the sum of the mutations in each mutation group of the non-treated group. For the statistical test, we performed one-sided Student’s *t*-test.

## Data Availability

All sequence reads data obtained in this study were deposited in the DDBJ under the accession number DRA016972.

## Abbreviations

TGR: Transgenic rodents
DEN: Diethylnitrosamine
PECC-Seq: Paired-end complementary consensus sequencing
CV: Coefficient of variation
